# Seasonal and Altitudinal Changes in Population Density of 20 Species of *Drosophila* in Chamundi Hill

**DOI:** 10.1673/031.010.12301

**Published:** 2010-08-03

**Authors:** Basavarajpur R. Guruprasad, Shridhar N. Hegde, Mysore S. Krishna

**Affiliations:** Department of Studies in Zoology, Manasagangotri, Mysore-570006, India

**Keywords:** cluster analysis, occurrence constancy method, Simpson Index, Berger-Parker Index

## Abstract

A year long study was conducted to analyze the altitudinal and seasonal variation in a population of *Drosophila* (Diptera: Drosophilidae) on Chamundi hill of Mysore, Karnataka State, India. A total of 16,671 *Drosophila* flies belonging to 20 species of 4 subgenera were collected at altitudes of 680 m, 780 m, 880 m and 980 m. The subgenus Sophophora was predominant with 14 species and the subgenus *Drosilopha* was least represented with only a single species. Cluster analysis and constancy methods were used to analyze the species occurrence qualitatively. Altitudinal changes in the population density, and relative abundance of the different species at different seasons were also studied. The diversity of the *Drosophila* community was assessed by applying the Simpson and Berger-Parker indices. At 680 m the Simpson Index was low at 0.129 and the Berger- Parker index was high at 1.1 at 980 m. Linear regression showed that the *Drosophila* community was positively correlated with rainfall but not elevation, Furthermore the density of *Drosophila* changed significantly in different seasons (F = 11.20, df 2, 9; P<0.004). The distributional pattern of a species or related group of species was uneven in space and time. *D. malerkotliana* and *D. nasuta* were found at all altitudes and can be considered as dominant species.

## Introduction

The family *Drosophilidae* (Diptera) is composed of more than 3,500 described species that occur in a number of ecosystems all over the world (Bachli 1998). Most genera are found in tropical regions. The *Drosophila* genus is the most abundant and comprises around 53% of the total species. Many of them are endemic to certain regions and a few are cosmopolitan, dispersed mostly in association with human activity. Studies of Drosophila have contributed to our understanding of principles of basic genetics, molecular biology, population genetics and evolution. *Drosophila* is also being used for the study of population fluctuations, as they are highly sensitive to slight environmental modifications that is reflected in the size of the natural population structure and ecology. It is known that changes in temperature and rainfall affect viability, fertility, developmental time and other factors that influence the rate of population growth and survival (Torres and Madi-Ravazzi 2006). Rainfall and light intensity also have an influence on the supply of resources, principally in relation to the periods of flowering and fruiting of various vegetable resources that provide most of the sites for oviposition and feeding (Brncic et al. 1985). In addition to above physical factors, biotic factors also influence the diversity and abundance of natural populations of *Drosophila* including intra—inter specific relationships, such as population density, population age, distribution, competition and relationship between *Drosophilids* and their hosts and predators. The number of the individuals of a species in a locality is significantly influenced by the presence or absence of another species, especially those that are ecologically related (Putman 1995; Begon 1996). The ability to colonize multiple niches is an indication of the biological success of many species (Torres and Madi-Ravazzi 2006).

Thus the presence or absence of a species in an ecological niche, and its richness or abundance in that area is an indicator of both biological and ecological diversity of that ecosystem. In addition to physical and biotic factors, the topography and season also affect the animal distribution. Elevation is one important aspect of topography and one has to look at the animal distribution from that perspective. A few attempts have been made to collect *Drosophila* at different altitudes, but these data are not analyzed with an ecological perspective (Reddy and Krishnamurthy 1977). Reddy and Krishnamurthy (1977) have also said that physical and biotic factors are the sole determinants of animal communities. If that is so elevation and season should not have any influence on animal distribution. In the present studies we propose to verify the effect of elevation and season on *Drosophila* community.

Furthermore, in the competitive exclusion theory, Gause suggested that two related species competing for the same resources could not co-exist together in the same ecological niche. Laboratory experiments have questioned the validity of the Gause Principle (Ayala 1969). The presence of taxonomically or phylogenetically related species in an ecological niche indicates their coexistence and absence of such related species suggests competitive exclusion. One aim of the present study is to investigate whether taxonomically or phylogenetically related *Drosophila* species co-exist in nature or not.

The present analysis of *Drosophila* community was done at different altitudes of Chamundi hill, Mysore (India). It is a small mountain (11′36′ N Latitude and 76′ 55′ E) with scrubby forest that was uninhabited about forty years ago with a small temple at the hilltop. However, the hill has become a famous tourist spot of Mysore (Karnataka, India) since about thirty years ago with a small township built at the top with a population of 2,000 and experiences the inflow of many tourists.

## Materials and Methods

The altitudinal and seasonal fluctuation in *Drosophila* fauna was studied in four different wild localities of Chamundi hill, Mysore. For this purpose monthly collection of flies were made at the altitudes of 680 m, 780 m, 880 m, and 980 m between February 2005 to January 2006. Both bottle trapping and net sweeping methods were used. For bottle trapping, milk bottles of 250 ml capacity containing smashed ripe banana sprayed with yeast were tied to the twigs underneath small bushes at a height of three to five feet above the ground. Five traps each were kept at each altitude. The following day the mouth of each bottle was plugged with cotton and removed from the bushes. The flies that were collected in the bottles were transferred to fresh bottles containing wheat cream agar medium (consisting 100 gm wheat powder, 120 gm raw sugar, 10 gm agar agar, 7 ml propionic acid boiled in 1000 ml water and cooled, Hegde et al, 2001) as food. Net sweeping was done on naturally rotting fruits if available or on fruits placed beneath shaded areas of the bushes one day before the collection. After each sweep, flies were transferred to the bottles containing fresh food. Five sweeps were made at each place so as to maintain uniformity in collection in each locality. The flies were brought to the laboratory, isolated, identified and sexed. Categorization of the collected *Drosophila* flies was made respective to taxonomic groups by employing several keys (Sturtevant 1927; Patterson and Stone 1952; Thorckmorton 1962; Bock 1971). To study seasonal variation the entire year was divided into three seasons; premonsoon extending from February-May, monsoon from June-September and post monsoon from October-January.

### Vegetation Collection sites

At 680 m: The foot of the hill was surrounded by mango orchards along with trees such as *Acacia concinna, Acacia catechu, Anacardium occidentale, Bombax ceiba, Breynea restusa, Cassia spectabilis, Celastrus paniculata, Cipadessa baccifera, Clematis trifolia, Dalbergia paniculata, Dioscorea pentaphylla, Ficus religiosa, Ficus bengalensis, Glyrecidia species,, Gymnima sylvestres, Hibiscus malva, Ichnocarpus frutescens, Lantana camera, Pongamia glabra, Phyllanthus species, Tamarindus indica, Thunbergia species, Tectona grandis, Sida retusa*, and many shrubs including cactus.

The vegetation both at 780 m and 880 m was the same. Major plants found in these localities were, *Albizzia amara, Andrographis serpellifolia, Argyria species, Bignonia species, Breynea restusa, Bridalia species, Cassia fistula, Cassine glauca, Eucalyptus grandis, Garcinia species, Lantana camera, Phyllanthus microphylla, Sida rhombifolia, Terminalia paniculata, Terminalia tomentosa, Vitex negundo, Zizipus oenoplea, Zizipus jujuba*.

The vegetation at the top of the hill (980 m) includes, *Acacia catechu, Anacardium occidentale, Autocarpus integrifolia, Jasminum species, Jatropa curcus, Lantana camera, Leus aspera, Mallotus philippensis, Murraya paniculata, Tamarindus indica, Zizipus jujuba*.

### Data Analyses

The relation between altitude, temperature, rainfall and density of flies was assessed through linear regression analysis keeping density as the dependent variable and temperature, altitude and rainfall as independent variables. The seasonal difference in population densities was studied by one-way analysis of variance (ANOVA) using SPSS 10.5. In order to verify the occurrence of a species qualitatively, the occurrence constancy method (Dijoz 1983) was used. The constancy value (c) was obtained by dividing the number of collections in which one species occurred by the total number of collections, and then multiplying that result by 100. Species with index c ≥ 50 were considered constants. Accessory species were those with 25 ≤ c < 50. Accidental species had c < 25. Species that occurred in only one area were considered exclusive. Cluster analysis as described by Mateus et al.(2006) and Giri et al. (2007) were used to design, analyze and compare different *Drosophila* populations on the hill. In the cluster study, Euclidean distance was chosen to measure the similarity between different species and Ward's Strategy (Giri et al. 2007) was followed to unite two clusters. A feature of Euclidean distance was that it is a weighted measurement; the higher the absolute value of the variable the higher will be its weight. *Drosophila* communities were analyzed using ecological indices including Simpson Berger-Parker, and Shannon-Wiener (Mateus et al. 2006).

The relationship between the abundance, richness and diversity of all groups of flies collected throughout the year was assessed by Simpson (D) and Berger-Parker (1/d) indices (Mateus *et al* 2006). The Shannon-Weiner index was also calculated, but the result was same as the Berger-Parkar index and was not included here. Among these, the Simpson index (D) that measures the probability that two individuals randomly selected from a sample that belong to the same species, was calculated using the formula,

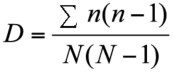

Where, n = the total number of organisms of a particular species and N = the total number of organisms of all population

Berger- Parker index (1/d) which shows the relative abundance was calculated using the formula,

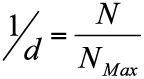

Where, N = Number of individuals of all species and N max = Number of individuals in the most common species

## Results

The distribution pattern of *Drosophila* species at four different altitudes of Chamundi hill is shown in Table 1. A total of twenty species were encountered in the hill that belonged to 4 subgenera namely *Sophophora, Drosophila, Dorsilopha, Scaptodrosophila*. Most of the species belonged to the *D. melanogaster* species group. *D. buskii* was the only species belonging to subgenus *Dorsilopha*. The total number of the flies captured through out the year was 16,671 and number of the species collected was 20. At 680 m, the number of flies collected was the highest (5,464) compared to all other altitudes and the least number was collected at 980 m. *D. nasuta, D. neonasuta, D. malerkotliana, D. rajasekari, D. jambulina, and D. bipectinata* were the most common species found at all altitudes compared to other species such as *D. anomelani, D. coonorensis, D. punjabiensis, D. mysorensis* and *D. gangotrii. D. kikkawii, D. takahashii, D. suzukii, D. repleta, D. immigrons, D. buskii, D. brindavani, D. nigra, D. mundagensis* were not found at all altitudes (Table 1).

The constancy value (c) of all species present at all altitudes along with absolute numbers (A) and relative abundance (r) are presented in Table 2. Constant species (c ≥ 50) represented approximately 72% of the total collected species (15 out of 20). Three species considered as accessory (18%) and 2 as accidental (10%) were found. *D. gangotrii, D. coonorensis,* considered as accessory species were found at 880 and 980 m but not found at 780 m and 680 m. All subgenera had constant species and the subgenus Sophophora had the most constant species (Table 2). The value of Simpson,and Berger-Parker indices that indicate the abundance, richness and diversity of *Drosophila* flies in different altitudes of the hill are given in Table 3. At the lowest altitude (680 m) Simpson = 0.129; and Berger-Parker = 1.05; and in higher altitude (980 m) Simpson was 0.15, Berger-Parker was 1.1,

**Figure 1.**
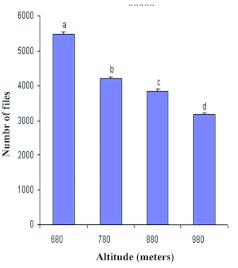
Altitudinal variation of *Drosophila* population at different altitudes of Chamundi hill m = meters (t = value: a = 3.471,b = 3.362,c = 3.112,d = 3.380: df = 19:p<0.01). High quality figures are available online.

**Figure 2.**
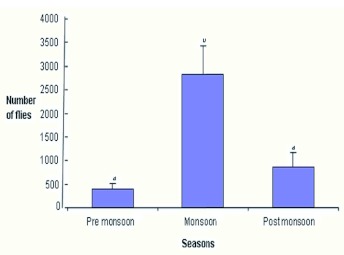
Seasonal variation in *Drosophila* flies collected from Chamundi hill (F = 11.20, df 2, 9; P<0.004). High quality figures are available online.

The number of *Drosophila* flies decreased with increasing altitude (Figure 1). The application of student t test between altitude and number of flies suggest that there was a significant difference in the population density of *Drosophila* at different altitudes. The seasonal variation in the population density of *Drosophila* is depicted in Figure 2. The density was low in pre-monsoon, increased in monsoon and again decreased in post-monsoon period. The analysis of variance calculated for pre-monsoon, monsoon and post-monsoon seasons showed significant differences between them (F = 11.20, df 2, 9, P<0.004). Table 4 shows the Linear regression analysis of temperature (r^2^ = 0.057; p = 0.1, f = 2.79), altitude (r^2^ = 0.025; p = 0.28, f = 1.18), rainfall (r^2^ = 0.333; p = 0.001, f = 23.0). There was negative correlation with altitude and temperature and positive correlation with rain.

**Figure 3.**
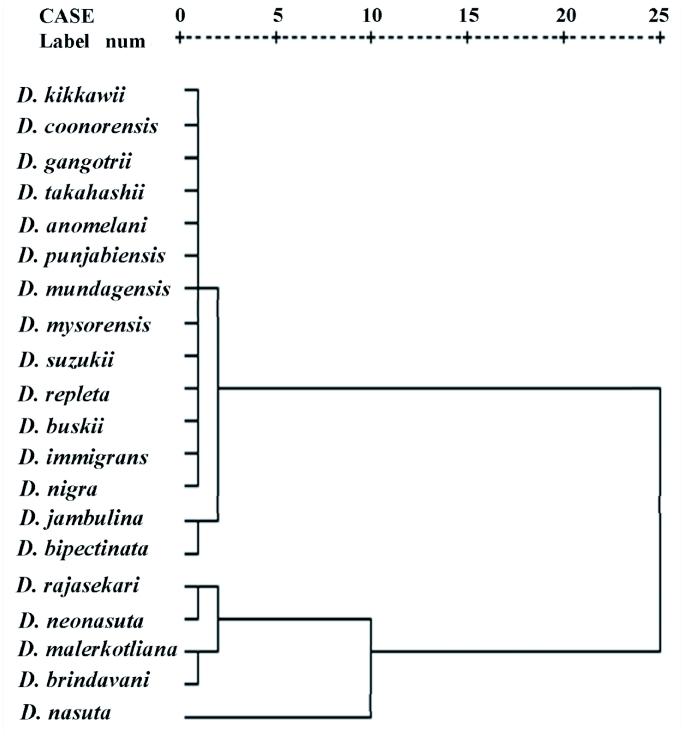
Dendrogram of *Drosophila* analyzed through cluster analysis. High quality figures are available online.

The cluster analysis performed on the basis of densities of different species showed two clusters (Figure 3). Of these two clusters, the first cluster belongs to *montium* sub group and included *D. kikkawii, D. coonorensis, D. gangotrii, D. takahashii, D. anomelani, D. punjabiensis, D. mundagensis, D. mysorensis* but *D. suzukii,* belongs to *suzukii* subgroup. Both these subgroups belong to the melanogaster species group of the subgenus *Sophophora. D. repleta, D. buskii,* and *D. immigrons* of the same cluster belong to subgenus *Drosophila,* while *D. nigra* belongs to subgenus *Scaptodrosophila. D. jambulina,* belongs to the *montium* subgroup and *D. bipectinata* belongs to the *ananassae* subgroup which is linked with the first cluster. In the second cluster, *D. rajasekari* belongs to *suzukii* subgroup of the *melanogaster* species group of subgenus *Sophophora* while *D. neonasuta* belongs to the subgenus *Drosophila. D. malerkotliana* and *D. brindavani* sub-cluster which joins with *D. rajasekari* and *D. neonasuta* belong to two different taxonomic categories. Among these, *D. malerkotliana* belongs to subgenus *Sophophora* and *D. brindavani* belongs to subgenus *Scaptodrosophila. D. nasuta* the lone third tier species which joins with the second cluster belong to the subgenus *Drosophila* and taxonomically more related to *D. neonasuta* of tier 1 species of this cluster. Thus most of the species of first cluster have closer taxonomic relationships than the second.

## Discussion

In the present studies the density of *Drosophila* at different altitudes of Chamundi hill decreased with increasing altitude (Table 1). At 680 m the density was highest and lowest at 980 m (Figure 1). The results indicate that *Drosophila* community is affected by elevation. Wakahama (1962) has reported similar altitudinal variation in the distribution of *Drosophila* in Mt.Dakesan in Japan. He found that total density decreases with increasing altitude. Reddy and Krishnamurthy (1977) have also noticed altitudinal variation in *Drosophila* populations in Jogimatti hills of Karnataka.

The regression analysis showed negative correlation with temperature and altitude and positive correlation with rain (Table 4). This suggests that the rainfall is one of the factors that affect *Drosophila* population density. The available reports on density of *Drosophila* are contradictory (Carson 1965; Reddy and Krishnamurty 1977). Some suggest that higher elevation is congenial and some suggest that lower elevation is congenial. The present study however clearly demonstrates that the altitude and other biotic and abiotic factors such as rain together determine the *Drosophila* community in a given ecosystem. The ecological conditions of Chamundi hill change with changing altitude, the lower altitude is comparatively cooler with lesser rain and dryness. Temperature and rain increase with increasing altitude except on the top of the hill.

**Table 1.**
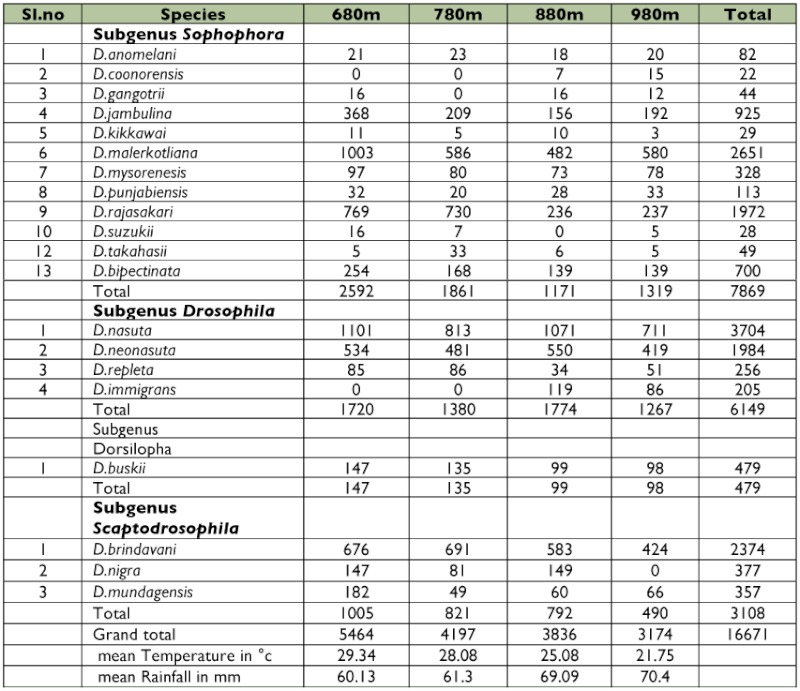
The *Drosophila* species and their numbers collected from the Chamundi hill during 2005–2006

According to Hegde et al. (2000) the growth and size of the population depend on several environmental factors in addition to genetic structure. Several earlier workers have been able to collect more flies of *D. nasuta* and *D. immigrons* at high altitudes than at low altitudes. These two species belong to the subgenus *Drosophila* and in the present study the authors collected 1,774 individuals of this subgenus at 880 m.

The fluctuation in population size of *Drosophila* through different seasons reflects the close relationship between population density with wet and dry seasons. Dobzhansky and Pavan (1950) showed that rainfall appears to have a greater influence on the abundance of *Drosophila* than temperature. In our study density was lowest during pre monsoon, which is the hot season, compared to monsoon season when rainfall increases. Population density declined from the middle of post monsoon when cold and dry weather prevail. There are number of factors that may influence the species richness of a community. They may be classified as 1) geographical (e.g. latitude and longitude); 2) environmental (an environment with a greater variety of niches would be able to host a greater variety of species); and 3) biological (the relationships of predation, competition and population density etc). These factors may have important consequences on the number of species in a given ecosystem. The changes in the natural environment caused by the alteration of seasons, would result in the change in relative frequency of different species from season to season (Figure 2). In tropical areas, especially in Brazil, changes in the environment are caused by the alteration between the dry and rainy seasons (Dobzhansky and Pavan 1950). It should be emphasized that the months with higher species richness occur during the rainy season. These differences suggest that at different altitudes the capacity to support *Drosophila* species varies. Thus the existence of seasonal variation in *Drosophila* species is quite evident by the presence of greater numbers of species in monsoon compared to pre and post monsoon periods. However, in temperate regions population densities decline to an extremely low level during cold winter months indicating the influence of temperature on the regulation of population size as is true in several *Drosophila* species inhabiting temperate regions (Patterson 1943; Dobzhansky and Pavan 1950; William and Miller 1952; Wakahama 1961). Thus it is evident that *Drosophila* community structure is affected by physical and biotic factors in addition to physiographic factors.

Table 2 shows that *D. anomelani, D. punjabiensis, D. repleta, D. immigrons, D. nigra, D. mundagensis, D. mysorensis, D. buskii, D. jambulina, D. bipectinata, D. nasuta, D. malerkotliana, D. rajasekari, D. brindavani and D. neonasuta* are constant species which are common in the hill. *D. coonorensis, D. gangotrii* and *D. takahashii are* accessory species while *D. kikkawii, D. suzukii,* are accidental species. In the cluster analysis, both accidental and accessory species occupy the first the cluster (Figure 3). Further in the first cluster, all species except *D. immigrons, D. buskii, D. nigra* and *D. repleta* are morphologically and phylogenetically related and hence they are classified in one subgenus Sophophora. The study therefore indicates the coexistence of species having similar ecological preferences supporting the view of Ayala (1969). Further in the second cluster, there are species belonging to different taxa, occupying different subclusters but joining with the main cluster at different tiers.

**Table 2.**
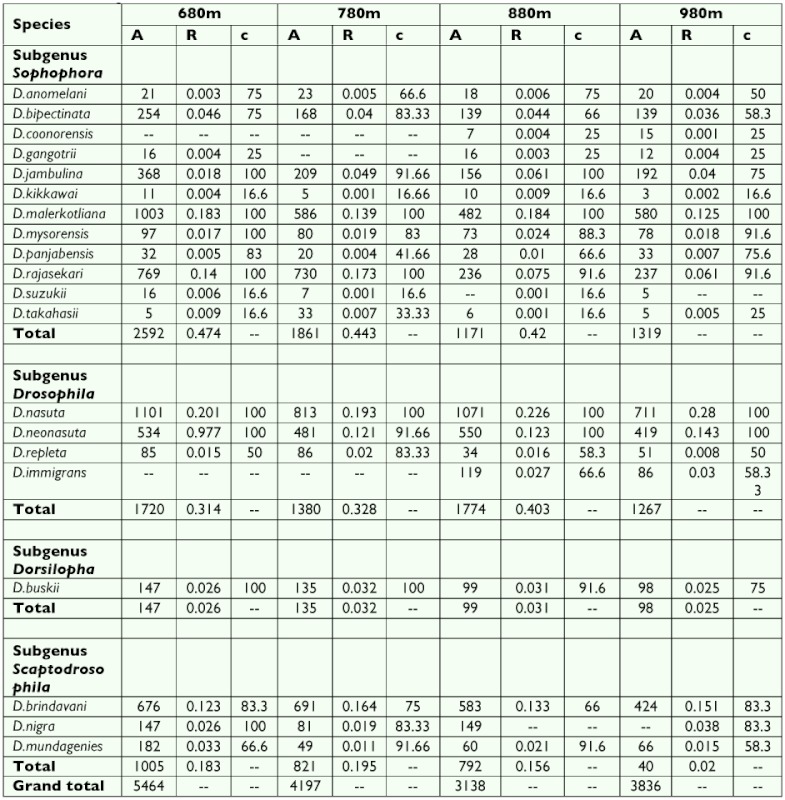
The absolute (A), relative abundance (R) and constancy value (c) Drosophila collected at the different altitudes of Chamundi hill during 2005–2006.

In the Simpson index (D) 0 represent infinite diversity and 1, no diversity, i.e, the greater the value of D the lower is the diversity but the reverse is true in case of Berger-Parker and Shannon-Wiener indices (Ludwig and Reynold, 1988 Mateus *et al* 2006). Applying these indices to understand the measures of biodiversity of flies at different altitudes of Chamundi hill demonstrates that the lower altitude of 680 m has a lower value (D) and higher value of 1/d indicating more biodiversity compared to the higher altitude of 980 m (Table 3). Although, these three indices revealed greater diversity at 680 m, more species were collected at 980 m. The reason for this may be easily understood if we observe the quantity and dominance of each species at each altitude, since the index combines two functions: number of species and uniformity, i.e. the number of individuals presented in each species (Ludwig and Reynold 1988; Torres and Madi-Ravazzi (2006). Again, this may be correlated to the vegetation and flowering plants at different altitudes. Thus, from the present ecodistributional analysis of *Drosophila* in Chamundi hill it is clear that the distributional pattern of a species or related group of species is uneven in space and time. *D. malerkotliana* and *D. nasuta* could be considered as dominant species, as they are registered in all altitudes with high numbers.

**Table 3.**
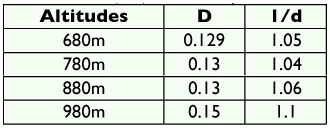
Simpson index (D) and Berger-Parker indices (l/d) for Drosophila collected at different altitudes of Chamundi hill.

**Table 4.**
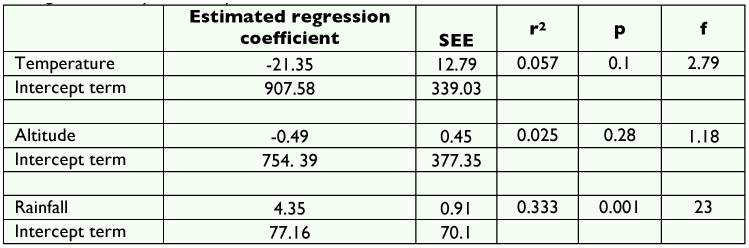
Linear regression analysis of temperature, altitude and rainfall.
